# NOTEWORTHY: THE IMPACT OF A CHOIR FOR OLDER ADULTS WITH DEMENTIA ON THE WELL-BEING AND QUALITY OF LIFE OF CAREGIVERS

**DOI:** 10.1093/geroni/igz038.2759

**Published:** 2019-11-08

**Authors:** Debra J Sheets, Stuart W MacDonald, Andre Smith, Mary Kennedy

**Affiliations:** University of Victoria, Victoria, British Columbia, Canada

## Abstract

Informal caregivers provide 80% of the care needed to support community-dwelling older adults with dementia. Over time caregivers often face adverse effects on their health, quality of life and well-being; particularly those caring for someone with dementia. This study examines the impact of participation in the Voices in Motion (ViM) choir on caregiver burden, mood and quality of life. A measurement burst approach was used to investigate intraindividual variability on key psychosocial and health indicators. Results indicate that choir participation significantly improves caregiver well-being (e.g. mood, burden) and quality of life. Findings suggest that choirs offer significant caregiver support and respite. The discussion focuses the public policy and on the potential economic implications which suggests a shift is needed in the services available to older adults with dementia and their caregivers.

